# Curcumin Intake Affects miRNA Signature in Murine Melanoma with mmu-miR-205-5p Most Significantly Altered

**DOI:** 10.1371/journal.pone.0081122

**Published:** 2013-12-12

**Authors:** Indra N. Dahmke, Christina Backes, Jeannette Rudzitis-Auth, Matthias W. Laschke, Petra Leidinger, Michael D. Menger, Eckart Meese, Ulrich Mahlknecht

**Affiliations:** 1 Institute for Clinical and Experimental Surgery, University of Saarland, Homburg/Saar, Germany; 2 Institute for Human Genetics, University of Saarland, Homburg/Saar, Germany; 3 Department for Internal Medicine - Hematology/Oncology, St. Lukas Klinik, Solingen, Germany; 4 Department of Internal Medicine, Division of Immunotherapy and Gene Therapy, Saarland University Medical Center, Homburg/Saar, Germany; University of Connecticut Health Center, United States of America

## Abstract

Melanoma is the most aggressive form of skin cancer with estimated 48,000 deaths per year worldwide. The polyphenol curcumin derived from the plant *Curcuma longa* is well known for its anti-inflammatory and anti-cancerogenic properties. Accordingly, dietary intake of this compound may be suitable for melanoma prevention. However, how this compound affects basic cellular mechanisms in developing melanoma still remains elusive. Therefore, the aim of this study was to investigate for the first time the impact of oral curcumin administration on the miRNA signature of engrafting melanoma. For this purpose, the effects of a 4% curcumin diet were tested on melanoma, which were established by injection of murine B78H1 cells in the flank of C57BL/6 mice. Curcumin diet or standard chow (control) was administered two weeks prior to injection of tumor cells until termination of the experiment. High throughput chip-based array analysis was deployed to detect alterations in the miRNA signature of the tumors. Curcumin treatment significantly reduced the growth of the flank tumors. Furthermore the miRNA expression signature in tumors was substantially altered by curcumin intake with mmu-miR-205-5p over 100 times higher expressed when compared to controls. The expression levels of identified key miRNAs in the tumor samples were confirmed by quantitative real-time polymerase chain reaction (qRT-PCR). A comparable expression pattern of these miRNAs was also detected in other curcumin-treated melanoma cell lines under *in vitro* conditions. Putative targets of curcumin-induced up-regulated miRNAs were enriched in ‘o-glycan biosynthesis’, ‘endoplasmatic reticulum protein processing’ and different cancer-related pathways. Western Blot analyses revealed that of these targets anti-apoptotic B-cell CLL/lymphoma 2 (Bcl-2) and proliferating cell nuclear antigen (PCNA) were significantly down-regulated in curcumin-treated tumors. These findings demonstrate a profound alteration of the miRNA expression signature in engrafting curcumin-treated melanoma with mmu-miR-205-5p being up-regulated most significantly.

## Introduction

Small non-coding RNAs, so-called microRNAs (miRNAs, miRs), post-transcriptionally attenuate many cellular processes [1], [2]. These evolutionary conserved miRNAs are about 22 nucleotides long and decrease protein expression in proliferating cells by binding to corresponding mRNAs leading either to transcriptional silencing or to mRNA degradation. Deregulation of the miRNA profile is found in many diseases including cancer [3]. For instance, miRNAs with anti-proliferative and anti-angiogenic properties such as hsa-miR-15, hsa-miR-16 or hsa-miR-221/-222 targeting vascular endothelial factor (VEGF), anti-B-cell CLL/lymphoma 2 (Bcl-2) and stem cell receptor c-kit, respectively, play an important role in either preventing or initiating carcinogenesis [4], [5], [6]. Because most mammalian mRNAs have been shown to be targeted by miRNAs, disease-associated expression changes are mirrored by changes of the miRNA profiles [7], [8]. As miRNAs can be purified from blood samples, it was suggested to utilize them as biomarkers for common illnesses, including cardiovascular diseases and cancer [9], [10]. Moreover, miRNA expression profiles may also be suitable as markers to evaluate the safety and efficacy of anti-cancer agents or to predict therapy response [11], [12], [13].

Melanoma is a highly metastatic skin cancer derived from malignant melanocytes. Since curing malignant melanoma remains difficult, evading risk factors is of upmost importance. Herein the avoidance of prolonged sun exposure and sunburns is most important and can be supported by additional dietary chemoprevention with green tea flavonoids, proanthocyanides, and vitamin E [14]. Also, the polyphenol curcumin (diferuloylmethan) derived from the rhizome of *Curcuma longa* has been thoroughly described for its chemopreventive effects by down-regulation of cellular pathways involved in protein-biosynthesis, mitochondrial activity and free radical scavenging [15], [16]. Oral administration of curcumin was shown to reduce skin inflammation, to support skin healing and even to suppress the development of chemically induced skin cancer in different animal models [17], [18], [19]. Besides, phase I and phase II clinical trials have demonstrated promising effects of oral curcumin administration in patients with colorectal neoplasia, advanced pancreatic and breast cancer either with or without additional chemotherapy [20], [21], [22], [23].

Recently, curcumin has been shown to influence miRNAs of different tumor entities, including pancreatic, breast and lung cancer cells [24], [25], [26]. For instance, down-regulation of *oncomir* hsa-miR-21 was found in colorectal cancer cells after stimulation with curcumin [27]. In addition, miRNAs from the hsa-let-7 and hsa-mir-200 families were up-regulated and hsa-miR-21 down-regulated by a synthetic curcumin derivate [28], [29].

In the present study we investigated the effects of oral curcumin intake on melanoma growth with the goal to identify potential changes of the tumor miRNA signature. We demonstrate a growth-inhibitory effect of dietary curcumin and profound changes in miRNA expression with mmu-miR-205-5p being up-regulated most significantly.

## Materials and Methods

### Curcumin

C3 complex, referred to in this article as curcumin, consisting of 77% curcumin, 17% demethoxycurcumin, and 3% bis-demethoxycurcumin was purchased in powdered form from Sabinsa Corporation.

### Ethics Statement

All animal care and experimental procedures were approved by the local governmental animal care committee (*Landesamt für Verbraucherschutz*, *Abteilung C Lebensmittel- und Veterinärwesen*, Saarbrücken, Germany; Permit Number: 07/2010) and were conducted in accordance with the European legislation on protection of animals (Guide line 2010/63/EU) and the NIH Guidelines for the Care and Use of Laboratory Animals (http://oacu.od.nih.gov/regs/index.htm. 8th Edition; 2011). All experiments were performed under isoflurane anesthesia, and all efforts were made to minimize animal suffering.

### Animals and curcumin diet

Male C57BL/6 mice with a body weight (b.w.) of 20-22g were used for the study and housed in groups of 3–4 animals. The animals were kept in a temperature- and humidity-controlled 12 h dark/light environment of the animal care facility of the Institute for Clinical and Experimental Surgery at the University of Saarland. They were allowed free access to tap water and animal chow (ssniff Spezialdiäten GmbH). The diet consisted of either standard mouse chow (control group) or chow enriched with 4% of curcumin (treatment group) prepared from the same batch. This equals a curcumin intake of 8 g/kg b.w. or 160 mg per day. The dietary intervention started 14 days before tumor induction and continued until the end of the *in vivo* experiments.

### Flank model

For continuous measurements of tumor size over 28 days, 1×10^5^ B78H1 cells were injected subcutaneously in each flank of male C57BL/6 mice (curcumin diet: n = 7, control: n = 6). B78H1 cells are murine amelanotic melanoma cells, which are Tap-2 and MHC-I negative clones derived from B16 cells in the laboratory of S. Silagi [30]. The B78H1 cells were a kind gift from the Laboratory of Immunology and Biology of Metastasis of the Department of Experimental Pathology at the University of Bologna [31]. Once a week tumor volumes were assessed by means of the high-resolution ultrasound imaging system Vevo 770 (VisualSonics, Inc.). For this purpose, the mice were anesthetized with 4% isoflurane (Baxter) and fixed in supine position on a heated stage. Anesthesia was maintained at 2% isoflurane and ultrasound coupling gel (Aquasonic 100; Parker Laboratories Inc.) was generously applied to the flanks before images were obtained. The stage was driven by a motorized mechanism so that the real-time microvisualization (RMV) 707B-scanhead (Visual Sonics; 30 MHz) was conducted linearly across the skin of the animals. To acquire parallel two-dimensional (2D) images, recording was done at regular spatial intervals, uniformly spaced at 100 µm over the visual tumor and its close vicinity, with a field of view of 17×17 mm^2^. A predefined parallel geometry of the 2D images allowed 3D image reconstructions and subsequent determination of tumor volumes. After killing the animals with an overdose of pentobarbital, tumors were extracted and divided up in samples for miRNA isolation and analyses, quantitative real-time polymerase chain reaction (qRT-PCR), Western Blotting and immunohistochemistry.

### miRNA isolation and analysis

Total RNA was isolated after phenol/guanidine-based lysis of 50 µg samples from flank tumors by silicamembrane-based purification with the miRNeasy Mini Kit (Qiagen) following the manufacturer's instructions. Isolated RNA was analyzed on mouse Sure Print G3 miRNA V17.0 microarray chips (8x60K; # G4859B) from Agilent Technologies based on miRBase, release 17.0, representing 1079 mouse miRNAs with 40 replicates [32]. Briefly, hybridization of Cyanine3-labeled miRNA samples to the high-definition arrays was carried out at 55°C for 20 h under constant rotation (20 rpm). Array-chips were scanned in a high resolution microarray scanner from Agilent Technologies using Scan Control 8.5.1 software, 3 microns resolution in double path mode. Signal intensity values were extracted from the raw data file using *feature extraction software* (Agilent Technologies). We used the computed *gTotalProbeSignal*, which is the average of all the background corrected signals for each replicated probe. Then we summed up the *gTotalProbeSignals* to calculate the total expression value for each miRNA per sample. Quantile normalization was applied to normalize expression values across the arrays using the *preprocessCore* package of the programming language *R*. We performed a log2 transformation of the data, which were then deposited in NCBI's Gene Expression Omnibus and are accessible through GEO Series accession number GSE47211 (http://www.ncbi.nlm.nih.gov/geo/query/acc.cgi?acc= GSE47211) [33]. For cluster analysis, we applied complete linkage hierarchical clustering using the Euclidian distance to compute the dissimilarity of miRNA (rows) and samples (columns) independently of each other. To compare expression values of miRNAs between the control and treatment groups, we applied the independent two-tailed *t*-test to find significantly deregulated miRNAs. The computed *P*-values were adjusted for multiple testing using the FDR (false discovery rate) approach by Benjamini and Hochberg [34]. MiRNAs with a FDR adjusted *P*-value smaller than 0.05 were considered statistically significant.

### Pathway analysis of miRNA

For *in silico* data mining predicted mouse miRNA targets were downloaded from miRDB (http://mirdb.org/miRDB/). Pathways putatively regulated by miRNAs which are altered by curcumin consumption were found by over-representation analysis (ORA). This was carried out by means of the online analysis-tool *GeneTrail* [35]. As test sets we used the targets of the up-regulated and the down-regulated miRNAs found differentially expressed comparing treatment and control group. As reference set we used the targets of all mouse miRNAs on the microarray. *GeneTrail* exhibited a search in disease relevant cellular pathways in the Kyoto Encyclopedia of Genes and Genomes (KEGG) for enrichment of putative miRNA targets [36]. The *P*-values for the KEGG pathways were FDR adjusted and considered significant if smaller than 0.05.

### qRT-PCR of tumor samples

For the validation of the miRNA microarray results, qRT-PCR was performed to analyze the expression of the four selected miRNAs mmu-miR-205-5p, mmu-miR-205-3p, mmu-miR-142-5p and mmu-miR-130b-3p using miScript PCR System (Qiagen). For this purpose, RNA of tumor samples was reversely transcribed into cDNA with the miScript II RT Kit (Qiagen). The samples were analyzed by StepOnePlus™ RT-PCR System (Applied Biosystems) with miScript primer assays (Qiagen), according to the instructions of the manufacturer. RNU6B was used as endogenous control.

### Western Blot analysis

To investigate the expression of putative targets of mmu-miR-205-5p, which was identified to be the most up-regulated miRNA under curcumin treatment, the whole protein fractions were purified from the organic phase of the phenol/guanidine-based RNA-isolation of four randomly chosen curcumin-treated and four control tumors. Then, 7.5 µg of protein were loaded per lane, separated discontinuously on 12% sodium dodecylsulfate polyacrylamide gels and transferred to nitrocellulose membranes (BioRad). After blocking of non-specific binding sites, membranes were incubated overnight at 4°C with a mouse monoclonal anti-Bcl-2 antibody (1∶1.000; Cell Signaling), a mouse monoclonal anti-proliferating cell nuclear antigen (PCNA) antibody (1∶1.000; DAKO) and a rabbit polyclonal anti-E2F1 antibody (1∶200; Santa Cruz) followed by the corresponding horseradish peroxidase (HRP)-conjugated secondary antibodies (1∶5.000; Dianova). Protein expression was visualized using luminol-enhanced chemiluminescence (ECL Plus; GE Healthcare) and autoradiography film (Hyperfilm; GE Healthcare) as specified by the manufacturer. Signals were densitometrically assessed with the gel analysis tool of the software *ImageJ*.

### Immunohistochemistry

Formalin-fixed specimens of curcumin-treated and control tumors were embedded in paraffin. To analyze the microvessel density of the tumors by immunohistochemical detection of the endothelial cell marker CD31, 2 µm-thick sections were cut and stained with a monoclonal rat anti-mouse CD31 antibody (1∶30; Dianova) as primary antibody followed by cyanin-3-coupled goat anti-rat IgG (1∶50; Dianova) as secondary antibody. Counterstaining of cell nuclei was performed with Hoechst (1∶500; Sigma-Aldrich). Subsequently, sections were examined using a BZ-8000 microscope (Keyence) for the quantitative analysis of the microvessel density within the tumors, given in mm^−2^.

### qRT-PCR of melanoma cell lines

To investigate whether the curcumin-induced expression pattern of the key miRNAs identified in the *in vivo* experiments is also transferable to other melanoma cell lines, murine B78H1 cells as well as human SK-MEL-28 (ATCC® HTB-72™) and MeWo cells (ATCC® HTB-65™) were treated with 20 µM curcumin or vehicle (0.1% DMSO; control) at 37°C and 5% CO_2_ for 48 h. Subsequently, the cells were harvested and the expression of mmu-miR-205-5p, mmu-miR-205-3p (or hsa-miR-205-3p for human cells), mmu-miR-142-5p and mmu-miR-130b-3p was analyzed by qRT-PCR, as described above.

### Flow cytometric analysis of B78H1 cells

To investigate the effect of curcumin treatment on proliferation and apoptosis of unsynchronized B78H1 cells, cell cycle analyses were performed by means of flow cytometry. For this purpose, B78H1 cells were treated with 20 µM and 40 µM curcumin or vehicle (0.1% DMSO; control) at 37°C and 5% CO_2_ for 48 h. Subsequently, the cells were fixed in methanol and stored at −20°C overnight. After thawing and washing, the cells were then treated with RNase A (1∶400, 10 mg/mL stock solution; Macherey-Nagel), stained with propidium iodide (1∶100, 1 mg/mL stock solution; Sigma-Aldrich) and analyzed by means of a FACScan™ (BD Biosystems). Four independent experiments were performed and each sample was analyzed in duplicate.

### Statistical analysis

Values are shown as mean ± standard error of the mean (SEM), as fold expression compared to control (qRT-PCR) or as relative expression (Western Blot analysis). Data were first analyzed for normal distribution and equal variance. Differences between two experimental groups were calculated by the unpaired Student's *t*-test. To test for time effects during the duration of the experiment within each experimental group, analysis of variance (ANOVA) for repeated measures was applied. The *post hoc* analyses included the correction of the α-error according to Bonferroni probabilities to compensate for multiple comparisons (SigmaStat; Jandel Corporation). A value of *P*<0.05 was considered statistically significant.

## Results

### Analysis of tumor growth and vascularization

**Figure 1:**
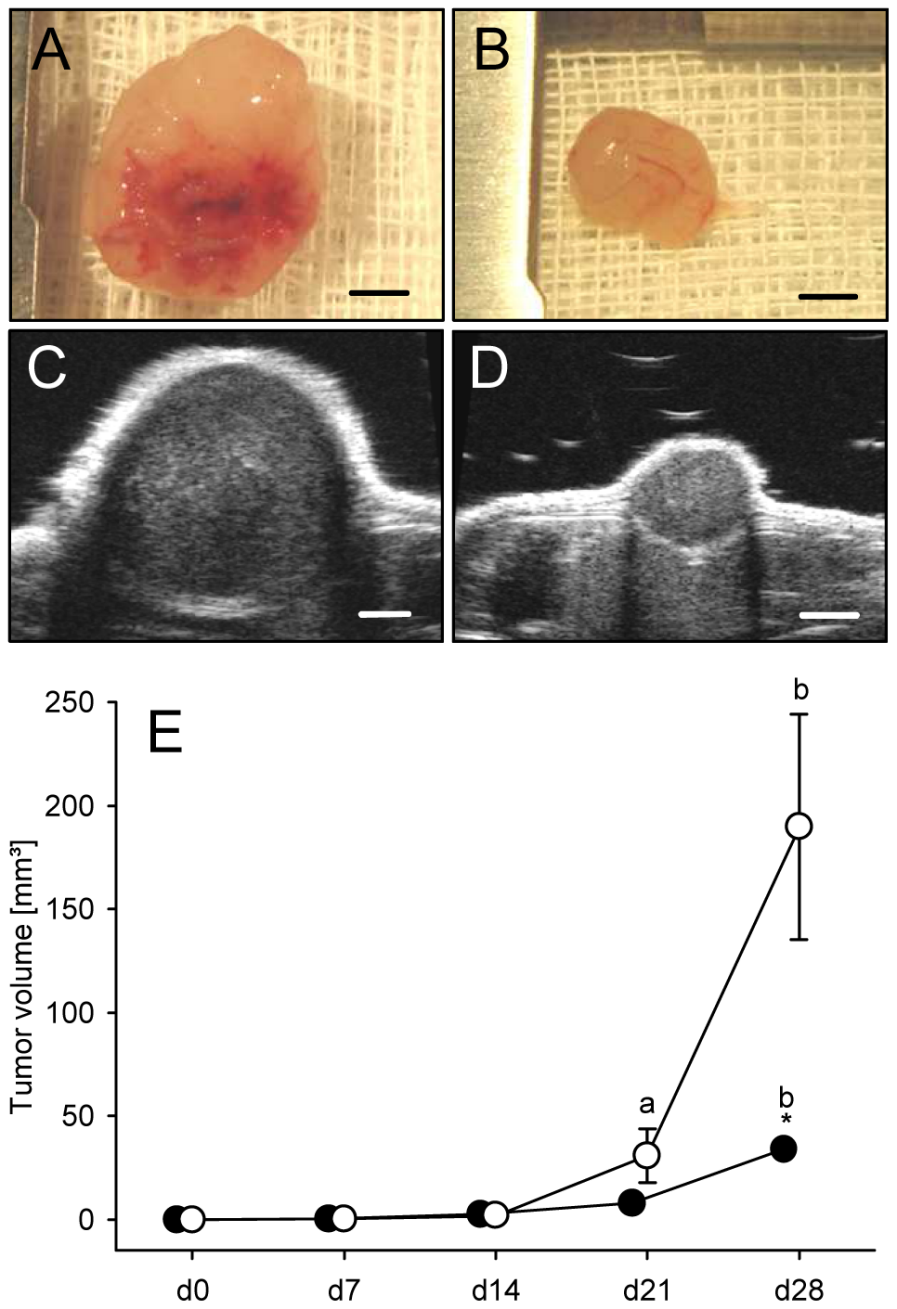
Significant growth inhibition of B78H1 melanoma by curcumin diet. A, B: Representative images of tumors from a control (A) and a curcumin-treated animal (B) at day 28. Scale bars: 2.7 mm. C, D: High resolution ultra-sound images of B78H1 tumors of either a control (C) or a curcumin-treated mouse (D) at day 28. Scale bars: 2.0 mm. E: The volume (mm^3^) of control tumors (white circles) and curcumin-treated tumors (black circles), as assessed by repetitive high-resolution ultrasound imaging. Means ± SEM. ^a^ *P*<0.05 vs. d0, d7 and d14 within the individual group; ^b^ *P*<0.05 vs. d0, d7, d14 and d21 within the individual group; *P = 0.008 vs. control tumors.

We analyzed the growth of amelanotic B78H1 tumors in the flanks of curcumin-treated and untreated C57BL/6 mice by means of high-resolution ultrasound imaging. For this purpose, we subcutaneously injected 1×10^5^ B78H1 cells in both flanks of male C57BL/6 mice for the measurement of tumor growth over a period of 28 days and to obtain tumor samples for miRNA profiling. Repetitive ultrasound imaging of the developing flank tumors revealed a markedly decreased tumor volume in curcumin-treated animals at day 28 when compared to untreated controls (Figure 1). However, additional immunohistochemical analyses showed that the density of CD31-positive microvessels in curcumin-treated tumors (64±9 mm^−2^) did not significantly differ from that of controls (82±8 mm^−2^; *P* = 0.142).

### Analysis of miRNA expression

**Figure 2:**
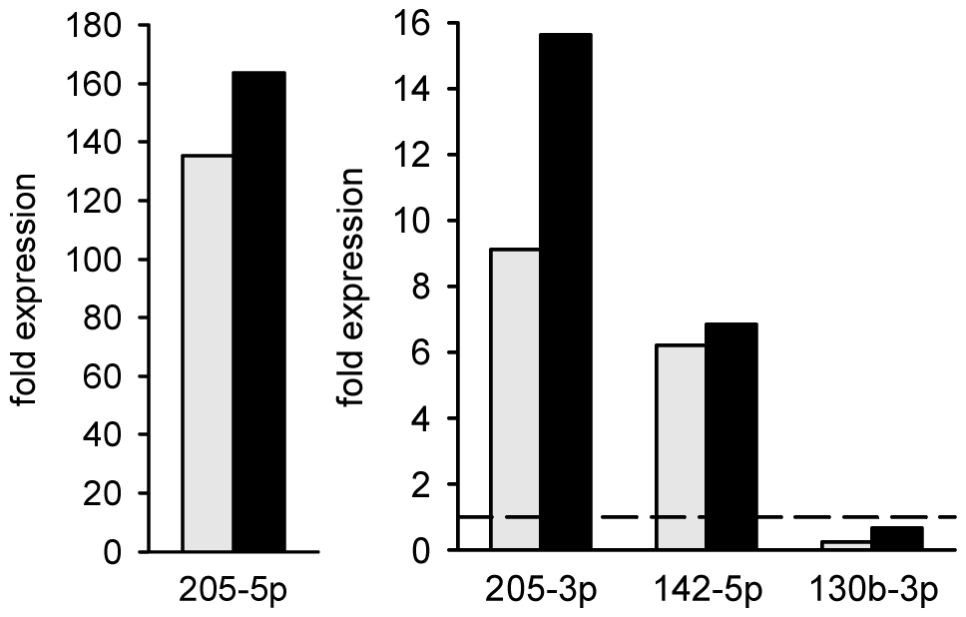
qRT-PCR validation of key miRNA expression in B78H1 melanoma regulated by curcumin diet. The diagrams display bar charts on the fold expression (compared to control) of mmu-miR-205-5p, mmu-miR-205-3p, mmu-miR-142-5p and mmu-miR-130b-3p in curcumin-treated B78H1 melanoma, as assessed by miRNA array (grey bars) and qRT-PCR (black bars). Broken line indicates expression level of control.

**Figure 3:**
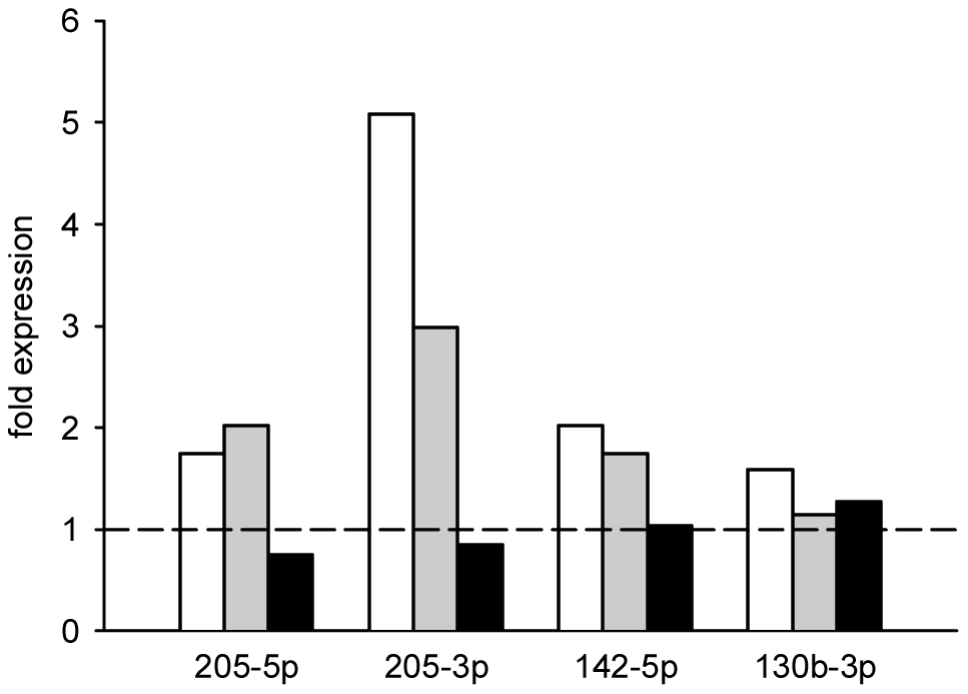
Expression of key miRNAs in different curcumin-treated melanoma cell lines. The diagram displays bar charts on the fold expression (compared to vehicle-treated control cells) of mmu-miR-205-5p, mmu-miR-205-3p, mmu-miR-142-5p and mmu-miR-130b-3p of curcumin-treated murine B78H1 (white bars), human SK-MEL-28 (grey bars) and human MeWo melanoma cells (black bars), as assessed by qRT-PCR. The cultured cells were treated with 20 µM curcumin or vehicle (0.1% DMSO) for 48 h. Broken line indicates expression level of control.

At day 28, total RNA including miRNA was extracted from flank tumors of curcumin-treated and untreated control animals. Following total RNA isolation from the flank tumors, we analyzed the expression of 1079 mouse miRNAs on a mouse Sure Print G3 miRNA V17.0 microarray from Agilent Technologies. We applied an independent two-tailed *t*-test to search for miRNAs that were significantly altered by curcumin intake. We identified 147 miRNAs to be significantly differentially regulated by curcumin administration with an adjusted *P*-value lower than 0.05. Out of the 86 up-regulated miRNAs, we found 49 miRNAs more than two-fold up-regulated, and out of the 61 down-regulated miRNAs, we found 34 miRNAs lower than 0.5-fold down-regulated (Table S1). The ten most up-regulated miRNAs included mmu-miR-205-5p, mmu-miR-222-3p, mmu-miR-205-3p, mmu-miR-146b-5p, mmu-miR-21-5p, mmu-miR-21-3p, mmu-miR-221-3p, mmu-miR-140-3p, mmu-miR-142-5p, and mmu-miR-140-5p and the ten most down-regulated miRNAs comprised mmu-miR-211-5p, mmu-miR-3096-5p, mmu-miR-711, mmu-miR-466h-5p, mmu-miR-130b-3p, mmu-miR-3082-5p, mmu-miR-1199-5p, mmu-miR-669b-5p, mmu-miR-1187, and mmu-miR-1224-5p (Table 1). The expression levels of mmu-miR-205-5p, mmu-miR-205-3p, mmu-miR-142-5p and mmu-miR-130b-3p, which have previously been described in the literature as miRNAs with potential anti-cancer properties [37], [38], were confirmed by qRT-PCR (Figure 2). Of interest, we also found these key miRNAs regulated by curcumin in cultured murine B78H1 cells and human SK-MEL-28 cells (Figure 3). In contrast, MeWo cells, which are derived from melanoma lymph node metastases, did not exhibit a strong regulation of these miRNAs (Figure 3).

**Table 1:** Top-ten miRNAs regulated by curcumin diet.

miRNA	Δ expression (fold change)	p vs. co (*t*-test)	sequence mmu	sequence hsa
**↑**				
205-5p	135.506	0.025	UCCUUCAUUCCACCGGAGUCUG	UCCUUCAUUCCACCGGAGUCUG
222-3p	9.392	0.003	AGCUACAUCUGGCUACUGGGU	AGCUACAUCUGGCUACUGGGU
205-3p	9.137	0.010	GAUUUCAGUGGAGUGAAG**CUCA**	GAUUUCAGUGGAGUGAAG**UUC**
146b-5p	7.379	0.005	UGAGAACUGAAUUCCAUAGGCU	UGAGAACUGAAUUCCAUAGGCU
21-5p	7.148	0.028	UAGCUUAUCAGACUGAUGUUGA	UAGCUUAUCAGACUGAUGUUGA
21-3p	6.877	0.003	CAACAGCAGUCGAUGGGCUGU**C**	CAACACCAGUCGAUGGGCUGU
221-3p	6.658	0.004	AGCUACAUUGUCUGCUGGGUUUC	AGCUACAUUGUCUGCUGGGUUUC
140-3p	6.647	0.003	UACCACAGGGUAGAACCACGG	UACCACAGGGUAGAACCACGG
142-5p	6.217	0.005	CAUAAAGUAGAAAGCACUACU	CAUAAAGUAGAAAGCACUACU
140-5p	5.524	0.004	CAGUGGUUUUACCCUAUGGUAG	CAGUGGUUUUACCCUAUGGUAG
**↓**				
211-5p	0.028	0.039	UUCCCUUUGUCAUCCUUUGCCU	UUCCCUUUGUCAUCCUUCGCCU
3096-5p	0.178	0.013	UGGCCAAGGAUGAGAACU	nd
711	0.222	0.016	GGGACCCGGGGAGAGAUGUAAG	nd
466h-5p	0.229	0.049	UGUGUGCAUGUGCUUGUGUGUA	nd
130b-3p	0.242	0.013	CAGUGCAAUGAUGAAAGGGCAU	CAGUGCAAUGAUGAAAGGGCAU
3082-5p	0.242	0.036	GACAGAGUGUGUGUGUCUGUGU	nd
1199-5p	0.261	0.023	UCUGAGUCCCGGUCGCGCGG	nd
669b-5p	0.294	0.049	AGUUUUGUGUGCAUGUGCAUGU	nd
1187	0.298	0.049	UAUGUGUGUGUGUAUGUGUGUAA	nd
1224-5p	0.299	0.010	GUGAGGACU**G**GGGAGGUGG**AG**	GUGAGGACU**C**GGGAGGUGG

The top-ten regulated miRNAs of B78H1 melanoma by curcumin diet are listed including the delta (Δ) fold change expression, *P*-values and sequences of both mouse (mmu) and human (hsa) species if available. Differences in the miRNA sequence between both species are indicated by bold letters. Note the high level of inter-species conservation of miRNAs.

**Figure 4:**
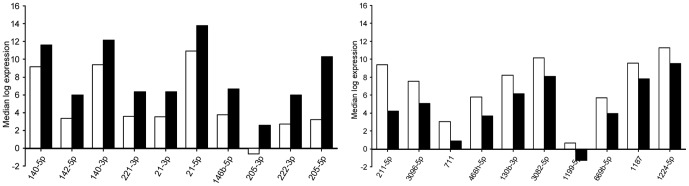
Expression profile of top-ten miRNA of B78H1 melanoma regulated by curcumin diet. The diagram displays bar charts on the median log expression of the top-ten up-regulated (A) and top-ten down-regulated (B) miRNA of the B78H1 melanoma after curcumin intake (black bars) compared to the control group (white bars).

**Figure 5:**
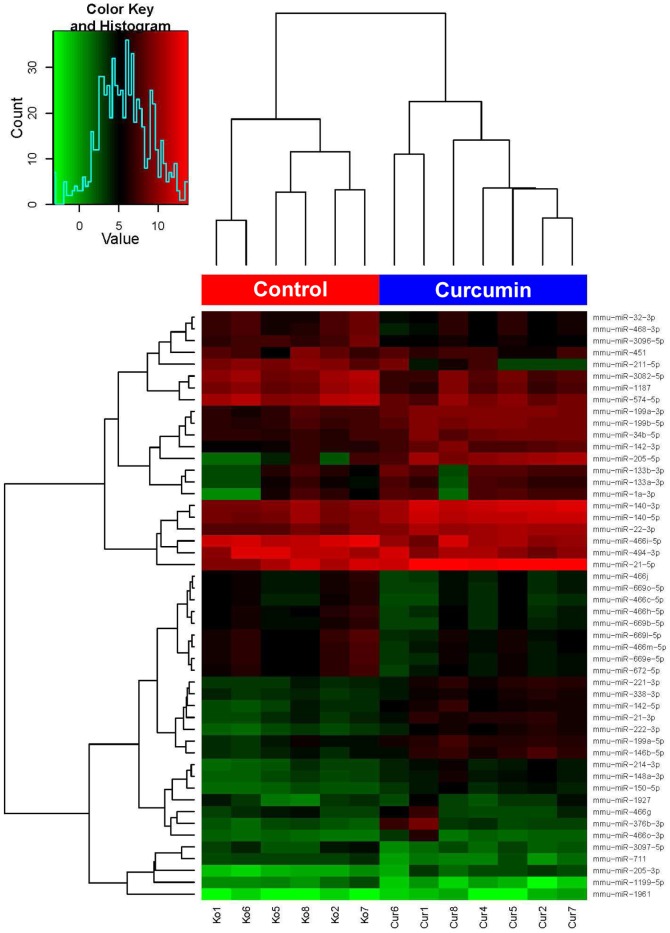
Curcumin diet causes clustering of biological replicates by changing B78H1 melanoma miRNA signature. Clustering of male C57BL/6 mice fed for 6 weeks either curcumin diet or control diet based on miRNA expression levels in the B78H1 melanoma. This diagram is based on 50 miRNAs with the highest variance across all samples. MiRNA expression variation is represented by different colors (see *color key*). Complete linkage hierarchical clustering was performed using the Euclidian distance to calculate the dissimilarity of miRNA and samples independently of one another. Clustering of samples is illustrated by upper dendrogram, clustering of miRNAs by the left dendrogram. Note that, based on the evaluated C57BL/6 mice, control and curcumin animals clearly divide into two main clusters.

The median log expression of the ten most deregulated miRNAs is given in Figure 4. The most up-regulated miRNA, mmu-mir-205-5p, was 135 times higher and the most down-regulated miRNA, mmu-mir-211-5p, was 35.7 times lower expressed in the curcumin-treated samples (Table 1). These data show that curcumin intake has a profound impact on the miRNA expression signature of the tumors. To further evaluate the effect of curcumin, we performed complete linkage hierarchical clustering using 50 miRNAs that showed the highest expression variance across all samples. As shown in Figure 5, we found two major clusters of miRNA expression profiles for 7 curcumin and 6 control samples. The obvious clustering between the two groups provides further evidence for the impact of the curcumin diet on the miRNA expression profile of the tumors.

Using our gene set analysis tool *GeneTrail*, we performed *in silico* pathway analyses separately for the predicted targets of the total of the 86 up-regulated and the total of 61 down-regulated miRNAs. Putative targets of curcumin-induced up-regulated miRNAs were enriched in the KEGG pathways “o-glycan biosynthesis”, “endoplasmatic reticulum protein processing” as well as in several cancer-related pathways (Table 2). Predicted targets of up-regulated miRNAs were also found to be enriched in the insulin-pathway and furthermore linked to heart-associated and neurological contexts (Table 2). Additionally, we detected over-representation in the Gene Ontology (GO) terms “cellular proliferation”, “cell death” and “regulation of apoptosis”. Predicted targets of down-regulated miRNAs were found to be enriched in a larger number of KEGG pathways including mTOR- and ErbB-signalling pathway, and also several cancer related pathways (Table 2).

**Table 2:** Pathways putatively regulated by curcumin-altered miRNAs as found by over-representation analysis.

	KEGG pathways with accumulated predicted targets	*P*-value	Expected number of targets	Observed number of targets	x-fold of target accumulation
Up-regulated miRNAs ↑					
	mucine type O-glycan biosynthesis	0.007	15	24	1.60
	renal cell carcinoma	0.024	37	50	1.35
	pathways in cancer	0.026	171	198	1.16
	protein processing in endoplasmatic reticulum	0.029	85	104	1.22
	acute myeloid leukemia	0.029	31	42	1.35
Down-regulated miRNAs ↓					
	Cholinergic synapsis	0.021	42	58	1.38
	ErbB signaling pathway	0.021	34	50	1.47
	Focal adhesion	0.021	80	102	1.28
	Neuroactive ligand-receptor interaction	0.021	94	117	1.24
	Pancreatic cancer	0.021	29	43	1.48
	Renal cell carcinoma	0.021	28	41	1.46
	Dilated cardiomyopathie	0.021	31	45	1.45
	Arrhythmogenic right ventricular cardiomyopathy	0.022	28	41	1.46
	Long-term potentiation	0.022	27	39	1.44
	Retrograde endocannabinoid sginaling	0.022	38	52	1.37
	Type II diabetes mellitus	0.022	18	28	1.56
	mTOR signaling pathway	0.022	26	38	1.47
	Hypertrophic cardiomyopathy (HCM)	0.025	30	42	1.42
	Prostate cancer	0.025	35	48	1.38
	Insulin signaling pathway	0.029	56	72	1.29
	Neurotrophin signaling pathway	0.030	48	63	1.31
	Morphine addiction	0.030	34	47	1.38
	Colorectal cancer	0.030	25	36	1.42
	Dopaminergic synapse	0.043	51	65	1.28

Listed are pathways being putatively regulated by miRNAs of B78H1 melanoma which are altered by curcumin diet, as found by over-representation analysis with the online analysis-tool *GeneTrail*. As test sets we used the predicted targets of differentially regulated miRNAs and as reference set the putative targets of all murine miRNAs printed on the microarray. *GeneTrail* exhibited a search in disease-relevant cellular pathways as found in the Kyoto Encyclopedia of Genes and Genomes (KEGG) for enrichment of putative targets. MiRNAs up-regulated by curcumin diet (upper part of table) were separately tested from down-regulated miRNAs (lower part of table). The expected number of target genes, modulated in a pathway, is shown as well as the observed ones, including the according *P*-value and x-fold enrichment of targets.

### Target validation

**Figure 6:**
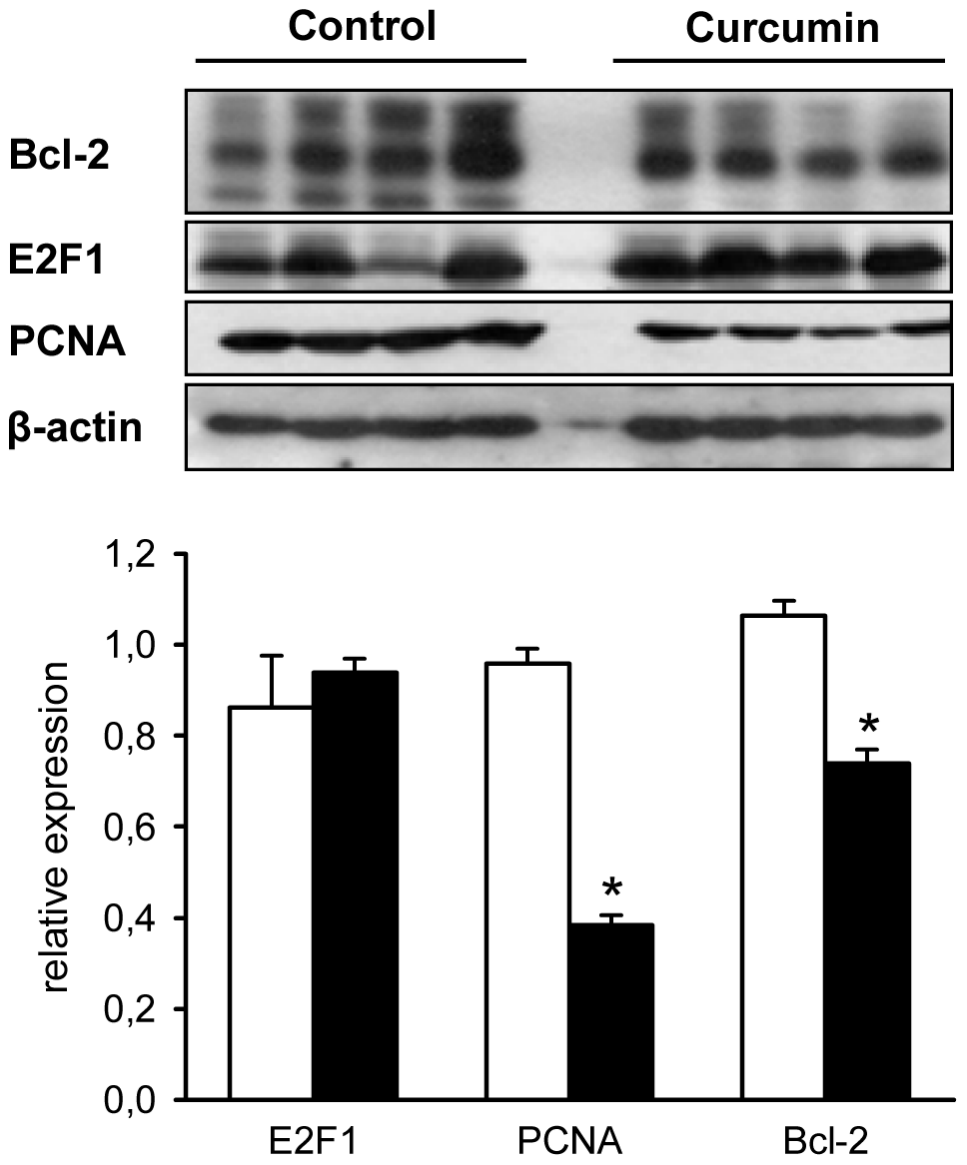
Western Blot analysis of the expression of target proteins of mmu-miR-205-5 in B78H1 melanoma. Relative expression of Bcl-2, E2F1 and PCNA in B78H1 melanoma of control (white bars, n = 4) and curcumin-treated animals (black bars, n = 4), as assessed by Western Blot analysis. Means ± SEM. *P<0.05 vs. control.

Based on our *in silico* analyses, we selected anti-apoptotic Bcl-2 and the transcription factor E2F1 for PCNA to validate some of the predicted targets of mmu-miR-205-5p, which was the most highly regulated miRNA by curcumin treatment. We found that Bcl-2 expression was significantly down-regulated in curcumin-treated tumors when compared to controls (Figure 6). In contrast, E2F1 expression was not markedly altered by curcumin treatment (Figure 6). However, the expression of the downstream proliferation marker PCNA was significantly reduced in the tumors under curcumin diet (Figure 6).

### Cell cycle analysis

**Figure 7:**
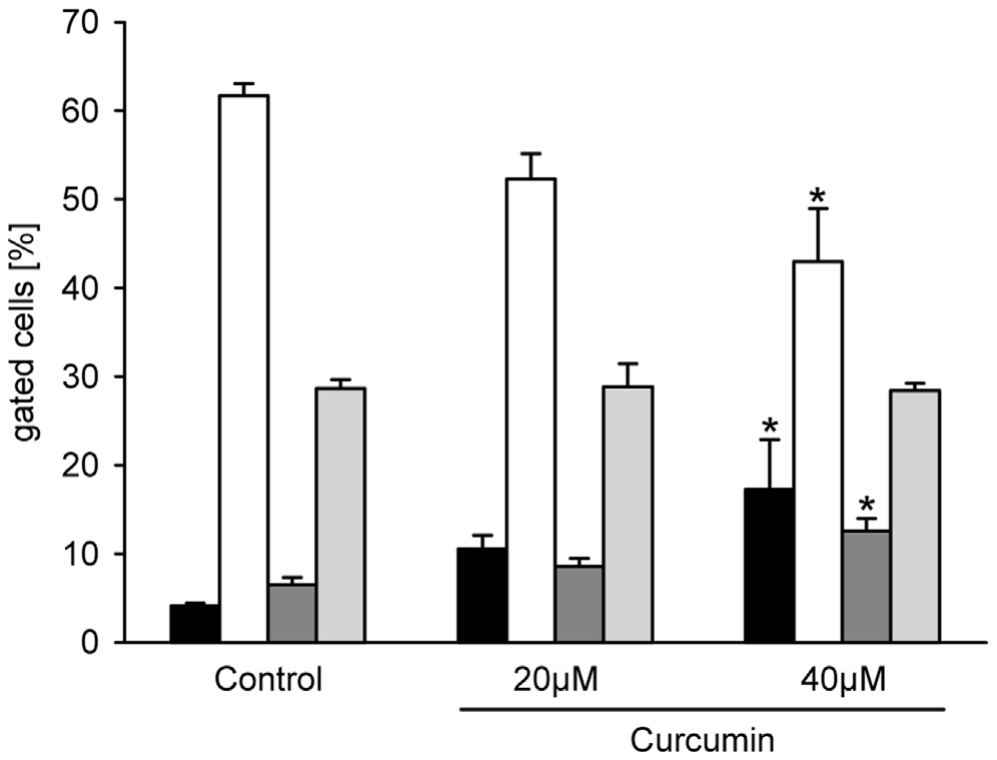
Cell cycle analysis of curcumin-treated B78H1 cells. Gated cells [%] in G1 phase (white bars), S phase (dark grey bars), G2 phase (light grey bars) and apoptosis (black bars), as measured by flow cytometry. The cultured cells were treated with 20 µM and 40 µM curcumin or vehicle (0.1% DMSO) for 48 h. Means ± SEM. *P<0.05 vs. control.

Flow cytometric cell cycle analyses showed dose-dependently a higher percentage of apoptotic cells in curcumin-treated B78H1 cells when compared to vehicle-treated controls (Figure 7). Moreover, curcumin treatment dose-dependently resulted in an S phase delay, indicating an additional inhibitory action on cell proliferation (Figure 7).

## Discussion

In the present study we investigated the effect of dietary curcumin consumption on the miRNA expression signature of engrafting mouse melanomas, which were established by injection of murine B78H1 cells in the flank of C57BL/6 mice. Because this approach does not include the transformation of normal cells into tumor cells (tumor initiation), the herein observed curcumin effects may be primarily of relevance for therapy or secondary prevention, but not for primary prevention of cancer.

We first analyzed potential anti-cancer properties of oral curcumin treatment that have previously been reported in the literature. These include in particular the inhibition of cell proliferation and tumor growth [18], [19], [39]. In line with these findings, we observed in our flank model a reduction of tumor size in curcumin-treated animals.

Moreover, curcumin has been described as an anti-angiogenic agent [16]. Therefore, we additionally measured the microvessel density of curcumin-treated and control tumors by immunohistochemical detection of the endothelial cell marker CD31. We found that the density of CD31-positive microvessels in curcumin-treated tumors did not significantly differ from that of controls. However, this does not necessarily indicate that curcumin did not exert an anti-angiogenic action on the treated tumors. In fact, according to Hlatky et al. [40] and Nico et al. [41], tumors under anti-angiogenic treatment may follow a ‘shrink to fit’ adaptation. This means that the tumor cell population may decrease in direct proportion to the loss of its supporting vasculature, resulting in a constant ratio between tumor cells and microvessels and, thus, a constant microvessel density. Accordingly, we herein found that tumor cell proliferation was markedly reduced in curcumin-treated animals when compared to controls.

In the next step we analyzed the miRNA signature of the isolated flank tumors, because there is increasing proof for miRNAs playing a pivotal role in the etiology of cancer. MiRNAs can act as tumor suppressors but also as oncogenes (*onco-mir*). Here, we provide strong evidence that the overall miRNA expression pattern in melanomas of curcumin-treated mice is markedly altered. The top ten miRNAs being significantly up-regulated by curcumin treatment comprised mmu-miR-205-5p (135-fold), mmu-miR-205-3p (9-fold) and mmu-miR-142-5p (6-fold). Their expression levels were confirmed by qRT-PCR. Up-regulation of mmu-miR-205-5p and -3p has been shown to reverse epithelial-to-mesenchymal transition in various tumors with E-cadherin as a central target [42], [43]. Hsa-miR-205 was found to be 100 times down-regulated in human metastatic melanoma compared to non-metastatic ones [38]. Down-regulation of miR-142-5p is associated with recurrence and poor overall survival in gastric and pancreatic cancer patients [12], [44]. Furthermore, we also identified several miRNAs, which were significantly down-regulated in curcumin-treated melanomas, such as mmu-miR-130b-3p. In former studies, this miRNA has been found to be up-regulated in cutaneous malignant melanoma [37]. Li et al. [45] could demonstrate that hsa-miR-130b induces epithelial-to-mesenchymal transition in endometrial cancer. Taken together, these results indicate that the curcumin-regulated miRNAs mmu-miR-205-5p, mmu-miR-205-3p, mmu-miR-142-5p and mmu-miR-130b-3p represent promising targets as well as markers to determine the aggressiveness and metastatic activity of malignant tumors.

In an additional experimental setting, we analyzed whether these key miRNAs underlie a similar regulation in different melanoma cell lines. For this purpose, we determined their expression by means of qRT-PCR in curcumin-treated and vehicle-treated murine B78H1 cells as well as human SK-MEL-28 and MeWo cells. Curcumin treatment resulted in higher expression levels in cultured B78H1 and SK-MEL-28 cells when compared to controls. In contrast, MeWo cells did not show a strong regulation of the key miRNAs. This may be due to the fact that MeWo cells are derived from melanoma lymph node metastases. Thus, they may exhibit a completely different miRNA expression profile when compared to cells originating from primary tumors.

Of interest, some of the most highly altered miRNAs were not regulated in a manner as expected based on the results of other studies. For example, down-regulation of hsa-miR-211-5p has been shown to be associated with melanoma progression [38], [46] and not as observed in the present study with inhibition of tumor growth in curcumin-treated melanoma. These contradictory findings may be explained by the fact that an altered miRNA expression does not necessarily imply a direct influence of curcumin on the miRNA expression of the tumor but can also be based on indirect feedback regulation. Furthermore, the effects of curcumin treatment may vary between tumors of different cell etiology or besides based on different experimental set-ups. For example, several investigators showed a down-regulation of up-regulated hsa-mir-21 after curcumin treatment in human pancreatic, oesophageal and prostate cancer cells [28], [29], [47], [48]. In contrast, Sun et al. [24] found an up-regulation of hsa-miR-21 expression induced by curcumin treatment in pancreatic cells similar to our findings.

Our over-representation analysis (ORA) identified several putative cellular pathways, which are modulated by up- and down-regulated miRNAs. Predicted targets of miRNAs were found to be enriched in metabolic pathways like insulin-pathway but also in heart-associated and neurological contexts, indicating a potential role for curcumin in managing metabolic syndrome. In addition, many of the pathways discovered by ORA were connected to cell proliferation and apoptotic cell death in cancer, emphasizing the impact of curcumin in the treatment of malignancies.

Based on the current literature and our *in silico* analyses, predicted targets of mmu-miR-205-5p include E2F1, E2F3, E2F5, Bcl-2, CD31, hypoxia-inducible transcription factor (HIF)-1α, and VEGF [49], [50]. From these targets we selected anti-apoptotic Bcl-2 and E2F1, which is a transcription factor for PCNA, for further validation by Western Blotting. Of interest, we found that Bcl-2 expression was significantly down-regulated in curcumin-treated tumors when compared to controls, whereas E2F1 expression was not markedly altered. The expression of PCNA, however, was significantly reduced under curcumin diet. This indicates that the expression of this proliferation marker may be regulated by other miRNAs than mmu-miR-205-5p or by other transcription factors than E2F1. In this context, mmu-miR-222 represents a potential candidate, which was also found to be up-regulated in the present study and directly regulates the expression of PCNA. Our results on the down-regulation of Bcl-2 and PCNA expression were further supported by flow cytometric cell cycle analyses of curcumin-treated B78H1 cells, indicating that curcumin affects both apoptosis and proliferation of this melanoma cell line.

In conclusion, we demonstrate for the first time a clear impact of dietary curcumin on the miRNA profile in murine melanoma with mmu-miR-205-5p being up-regulated most extensively. Linkage hierarchical clustering of the 50 miRNAs with the highest expression variance revealed a grouping into two main clusters, one comprising the treated animals and the other the controls. Putative targets of curcumin-induced up-regulated miRNAs are enriched in several cancer-related pathways. These data suggest miRNAs playing a significant role in melanoma growth inhibition by oral curcumin administration.

## Supporting Information

Table S1 ***Significant differential expression of miRNA after curcumin diet*** **(DOC).**
(DOCX)
