# Supplementary material for: Curcumin Intake Affects miRNA Signature in Murine Melanoma with mmu-miR-205-5p Most Significantly Altered
Source: PLoS One. 2013 Dec 12;8(12):e81122. doi: 10.1371/journal.pone.0081122 (PMC3861310; doi:10.1371/journal.pone.0081122)
Supplement: Table S1 — Significant differential expression of miRNA after curcumin diet (DOC). (DOCX) [file pone.0081122.s001.docx]

**Table S1.** Significant differential expression of miRNA after curcumin diet

|  | | | | | | |
| --- | --- | --- | --- | --- | --- | --- |
| **miRNA** | **median curcumin** | **median control** | **Δ log median** | ***t*-test adjp** | **AUC** | **fold change median** |
| mmu-miR-211-5p | 4,212295649 | 9,370758959 | -5,158463311 | 0,039156015 | 0,928571429 | 0,027999341 |
| mmu-miR-3096-5p | 5,063135797 | 7,549351952 | -2,486216156 | 0,013263707 | 1 | 0,178473755 |
| mmu-miR-711 | 0,893675241 | 3,067677242 | -2,174002001 | 0,016204944 | 0,976190476 | 0,221595118 |
| mmu-miR-466h-5p | 3,671177897 | 5,799445171 | -2,128267274 | 0,048840193 | 0,904761905 | 0,228732413 |
| mmu-miR-130b-3p | 6,16555455 | 8,209973701 | -2,044419151 | 0,013263707 | 0,952380952 | 0,242420036 |
| mmu-miR-3082-5p | 8,101702315 | 10,14600945 | -2,044307138 | 0,036251627 | 0,928571429 | 0,242438859 |
| mmu-miR-1199-5p | -1,291773774 | 0,645855229 | -1,937629003 | 0,023252834 | 1 | 0,261045102 |
| mmu-miR-669b-5p | 3,932993483 | 5,70061625 | -1,767622767 | 0,048840193 | 0,904761905 | 0,293692277 |
| mmu-miR-1187 | 7,819397498 | 9,564258891 | -1,744861393 | 0,048840193 | 0,904761905 | 0,2983626 |
| mmu-miR-1224-5p | 9,522162446 | 11,2659021 | -1,743739653 | 0,010251777 | 0,976190476 | 0,298594676 |
| mmu-miR-301b-3p | 2,561195207 | 4,278877606 | -1,717682399 | 0,007669065 | 0,976190476 | 0,304036745 |
| mmu-miR-574-5p | 9,180611 | 10,83080124 | -1,650190237 | 0,039529506 | 0,928571429 | 0,318598143 |
| mmu-miR-18a-5p | 6,327451498 | 7,948986574 | -1,621535076 | 0,008707755 | 0,952380952 | 0,32498948 |
| mmu-miR-3057-5p | 1,309813159 | 2,878867419 | -1,56905426 | 0,014681752 | 1 | 0,337029257 |
| mmu-miR-5109 | 11,4795924 | 13,03766425 | -1,558071854 | 0,019061027 | 0,94047619 | 0,339604657 |
| mmu-miR-2137 | 6,910623364 | 8,411315353 | -1,500691989 | 0,026362098 | 1 | 0,353383849 |
| mmu-miR-5103 | 2,869047759 | 4,36104693 | -1,491999171 | 0,023854625 | 1 | 0,355519557 |
| mmu-miR-3096b-5p | 3,706199191 | 5,149430112 | -1,443230921 | 0,016293665 | 1 | 0,36774282 |
| mmu-miR-20a-5p | 9,093092085 | 10,50454569 | -1,4114536 | 0,025208325 | 0,976190476 | 0,375932722 |
| mmu-miR-362-5p | 3,881599974 | 5,234012112 | -1,352412138 | 0,021298174 | 0,952380952 | 0,391636698 |
| mmu-miR-2861 | 8,165013957 | 9,49970614 | -1,334692183 | 0,019061027 | 1 | 0,396476653 |
| mmu-miR-188-5p | 6,910623364 | 8,226575638 | -1,315952274 | 0,027281328 | 1 | 0,401660286 |
| mmu-miR-5097 | 10,51192014 | 11,7985183 | -1,286598161 | 0,019061027 | 0,928571429 | 0,409916464 |
| mmu-miR-5105 | 8,209973701 | 9,455493373 | -1,245519672 | 0,036450658 | 0,904761905 | 0,42175595 |
| mmu-miR-714 | 4,084823064 | 5,322991558 | -1,238168494 | 0,023252834 | 0,952380952 | 0,42391047 |
| mmu-miR-532-5p | 6,055866127 | 7,279528264 | -1,223662137 | 0,025041295 | 0,952380952 | 0,428194409 |
| mmu-miR-2182 | 2,510442485 | 3,714904508 | -1,204462023 | 0,003593691 | 1 | 0,433931125 |
| mmu-miR-19b-3p | 9,093092085 | 10,24854019 | -1,155448102 | 0,010251777 | 0,976190476 | 0,448926728 |
| mmu-miR-1306-3p | 4,156151292 | 5,288414333 | -1,132263041 | 0,02201819 | 0,952380952 | 0,45619956 |
| mmu-miR-129-5p | 1,322403884 | 2,384524513 | -1,062120629 | 0,014681752 | 0,976190476 | 0,478927562 |
| mmu-miR-362-3p | 5,946216628 | 7,001973035 | -1,055756408 | 0,009756727 | 0,952380952 | 0,481044943 |
| mmu-miR-326-3p | 3,79540551 | 4,818940321 | -1,023534812 | 0,0265912 | 0,952380952 | 0,491909625 |
| mmu-miR-128-3p | 5,288460545 | 6,310591221 | -1,022130677 | 0,021790703 | 0,952380952 | 0,49238862 |
| mmu-miR-20b-5p | 7,675398788 | 8,683360157 | -1,007961369 | 0,016565914 | 0,952380952 | 0,497248399 |
| mmu-miR-3960 | 10,12962072 | 11,12246036 | -0,992839637 | 0,03781209 | 0,880952381 | 0,502487761 |
| mmu-miR-19a-3p | 6,34088958 | 7,314962178 | -0,974072598 | 0,005199678 | 0,976190476 | 0,509066983 |
| mmu-miR-680 | 4,889868024 | 5,788514743 | -0,898646719 | 0,019061027 | 0,952380952 | 0,536389641 |
| mmu-miR-5131 | 3,918729135 | 4,8107285 | -0,891999365 | 0,025041295 | 1 | 0,53886681 |
| mmu-miR-5126 | 11,4795924 | 12,34261101 | -0,863018605 | 0,046900264 | 0,916666667 | 0,549800984 |
| mmu-miR-3095-3p | 3,120663018 | 3,920766581 | -0,800103563 | 0,01350172 | 1 | 0,57430795 |
| mmu-miR-25-3p | 8,135418016 | 8,927407587 | -0,791989571 | 0,025754732 | 0,928571429 | 0,577547067 |
| mmu-miR-324-5p | 6,783376668 | 7,552941267 | -0,7695646 | 0,027771154 | 0,964285714 | 0,58659448 |
| mmu-miR-1901 | 3,201650604 | 3,960890862 | -0,759240258 | 0,03897942 | 0,94047619 | 0,590807376 |
| mmu-miR-500-3p | 7,373304483 | 8,118560166 | -0,745255683 | 0,011103822 | 0,952380952 | 0,596562136 |
| mmu-miR-351-3p | 4,264307901 | 4,996046918 | -0,731739017 | 0,014860474 | 0,988095238 | 0,602177614 |
| mmu-miR-130a-3p | 10,01071074 | 10,70229396 | -0,69158322 | 0,014681752 | 0,964285714 | 0,619173993 |
| mmu-miR-1982-5p | 5,509925484 | 6,191037692 | -0,681112209 | 0,042679124 | 1 | 0,623684276 |
| mmu-miR-187-5p | 1,53600074 | 2,184932889 | -0,648932149 | 0,014681752 | 1 | 0,637752189 |
| mmu-miR-483-5p | 4,636411771 | 5,284078643 | -0,647666872 | 0,019728756 | 1 | 0,638311758 |
| mmu-miR-804 | 2,883574696 | 3,519136306 | -0,63556161 | 0,025208325 | 0,976190476 | 0,643690191 |
| mmu-miR-290-5p | 4,375728305 | 5,00594537 | -0,630217064 | 0,047108649 | 0,869047619 | 0,6460792 |
| mmu-miR-760-5p | 2,15019381 | 2,777090092 | -0,626896282 | 0,020234346 | 1 | 0,647568053 |
| mmu-miR-1249-3p | 5,729095519 | 6,337815611 | -0,608720092 | 0,043871348 | 0,880952381 | 0,655778227 |
| mmu-miR-712-3p | 1,551594994 | 2,151457328 | -0,599862334 | 0,042523743 | 1 | 0,659816914 |
| mmu-miR-92a-3p | 8,135418016 | 8,722630951 | -0,587212934 | 0,041227762 | 0,892857143 | 0,665627556 |
| mmu-miR-210-3p | 8,807562271 | 9,363448746 | -0,555886475 | 0,033252244 | 0,988095238 | 0,680238952 |
| mmu-miR-1965 | 1,369419966 | 1,896495144 | -0,527075178 | 0,029191842 | 0,976190476 | 0,693960197 |
| mmu-miR-5120 | 2,448587042 | 2,954231107 | -0,505644065 | 0,033635266 | 0,952380952 | 0,704345865 |
| mmu-miR-31-3p | 7,347974273 | 7,768730235 | -0,420755962 | 0,025208325 | 0,976190476 | 0,747033082 |
| mmu-miR-3060-5p | 1,829025936 | 2,226940376 | -0,397914439 | 0,048840193 | 0,928571429 | 0,758954636 |
| mmu-miR-483-3p | 4,357096626 | 4,701556784 | -0,344460158 | 0,019061027 | 1 | 0,787602634 |
| mmu-miR-875-5p | 1,937604769 | 1,758266387 | 0,179338383 | 0,04690671 | 0,023809524 | 1,132364466 |
| mmu-miR-872-3p | 2,788823941 | 2,501359922 | 0,28746402 | 0,033533216 | 0,047619048 | 1,220492999 |
| mmu-miR-409-5p | 2,142796806 | 1,840911789 | 0,301885017 | 0,046397353 | 0,023809524 | 1,232754071 |
| mmu-miR-450a-1-3p | 1,366839248 | 1,041756694 | 0,325082554 | 0,04690671 | 0,047619048 | 1,25273612 |
| mmu-miR-152-5p | 1,784434443 | 1,43720189 | 0,347232553 | 0,043871348 | 0,095238095 | 1,272118047 |
| mmu-miR-191-5p | 1,84910729 | 1,485951626 | 0,363155664 | 0,033074216 | 0,023809524 | 1,286236258 |
| mmu-miR-190-5p | 2,128107863 | 1,747660063 | 0,3804478 | 0,019061027 | 0,023809524 | 1,301745844 |
| mmu-miR-100-3p | 2,300734978 | 1,903241552 | 0,397493426 | 0,014289986 | 0 | 1,317217356 |
| mmu-miR-29a-5p | 2,666735414 | 2,250659777 | 0,416075637 | 0,025564393 | 0,035714286 | 1,334293122 |
| mmu-miR-23a-3p | 11,24457203 | 10,81153727 | 0,433034761 | 0,019061027 | 0,047619048 | 1,350070514 |
| mmu-miR-181c-5p | 3,027459327 | 2,550654293 | 0,476805034 | 0,043967924 | 0,047619048 | 1,39165831 |
| mmu-miR-23b-5p | 1,183575391 | 0,689180092 | 0,494395298 | 0,006152604 | 0 | 1,408730166 |
| mmu-miR-135b-5p | 2,974256448 | 2,452835188 | 0,521421261 | 0,008254166 | 0 | 1,435368595 |
| mmu-miR-154-3p | 2,648186111 | 2,104547301 | 0,543638809 | 0,030171685 | 0 | 1,4576444 |
| mmu-miR-320-3p | 5,968250638 | 5,41218159 | 0,556069049 | 0,039529506 | 0,071428571 | 1,470257702 |
| mmu-miR-224-5p | 2,503971986 | 1,945543762 | 0,558428224 | 0,016193578 | 0 | 1,472663917 |
| mmu-miR-351-5p | 3,784150425 | 3,193338854 | 0,590811571 | 0,047075555 | 0,047619048 | 1,506093745 |
| mmu-miR-350-3p | 7,185752046 | 6,563758737 | 0,621993309 | 0,043935282 | 0,119047619 | 1,539000082 |
| mmu-miR-139-5p | 2,841929672 | 2,216191244 | 0,625738428 | 0,021733239 | 0 | 1,543000392 |
| mmu-miR-411-5p | 2,874749066 | 2,189385632 | 0,685363434 | 0,042679124 | 0,023809524 | 1,608107034 |
| mmu-miR-28c | 3,339998665 | 2,645674662 | 0,694324003 | 0,014681752 | 0,047619048 | 1,618126058 |
| mmu-miR-802-5p | 2,892109255 | 2,192706387 | 0,699402869 | 0,016204944 | 0 | 1,623832549 |
| mmu-miR-125a-5p | 7,904199775 | 7,196256322 | 0,707943453 | 0,012610473 | 0 | 1,633473957 |
| mmu-miR-3068-3p | 3,680057339 | 2,888302081 | 0,791755258 | 0,028400474 | 0,011904762 | 1,731179425 |
| mmu-miR-335-5p | 3,004620869 | 2,189229738 | 0,81539113 | 0,010251777 | 0 | 1,759775181 |
| mmu-miR-146a-5p | 6,945537841 | 6,124326046 | 0,821211795 | 0,026826396 | 0 | 1,766889472 |
| mmu-miR-27b-3p | 9,614127398 | 8,786957275 | 0,827170123 | 0,016565914 | 0,023809524 | 1,774201812 |
| mmu-miR-151-5p | 9,164534006 | 8,300283081 | 0,864250925 | 0,014596753 | 0,011904762 | 1,820394238 |
| mmu-miR-99b-5p | 7,206760598 | 6,317236674 | 0,889523925 | 0,008361447 | 0,011904762 | 1,852564695 |
| mmu-miR-455-5p | 3,900291168 | 2,999084799 | 0,901206368 | 0,022020118 | 0,071428571 | 1,867627023 |
| mmu-miR-542-3p | 5,063135797 | 4,156639959 | 0,906495838 | 0,0348842 | 0,119047619 | 1,874487024 |
| mmu-miR-223-3p | 7,347974273 | 6,404744766 | 0,943229507 | 0,031412382 | 0 | 1,922827719 |
| mmu-let-7e-5p | 9,015563894 | 8,058024781 | 0,957539112 | 0,014681752 | 0,047619048 | 1,941994497 |
| mmu-miR-218-5p | 3,01211159 | 2,050296027 | 0,961815563 | 0,003593691 | 0 | 1,947759516 |
| mmu-miR-24-2-5p | 5,904741829 | 4,939183923 | 0,965557907 | 0,016204944 | 0,047619048 | 1,952818553 |
| mmu-miR-425-5p | 6,192935681 | 5,223294106 | 0,969641575 | 0,006668949 | 0 | 1,958353999 |
| mmu-miR-219-5p | 5,369967064 | 4,394375668 | 0,975591396 | 0,04032174 | 0,095238095 | 1,966447127 |
| mmu-miR-26a-5p | 10,51192014 | 9,49970614 | 1,012214003 | 0,005199678 | 0,011904762 | 2,017004081 |
| mmu-miR-450b-3p | 4,273412736 | 3,259477674 | 1,013935062 | 0,049633461 | 0,119047619 | 2,019411697 |
| mmu-miR-222-5p | 2,364736022 | 1,35030469 | 1,014431332 | 0,014681752 | 0 | 2,020106469 |
| mmu-miR-24-1-5p | 4,686689645 | 3,603166089 | 1,083523556 | 0,021733239 | 0,047619048 | 2,119205592 |
| mmu-miR-26b-5p | 8,549066719 | 7,434951298 | 1,11411542 | 0,010251777 | 0,05952381 | 2,164622459 |
| mmu-miR-193b-3p | 3,325676492 | 2,168187918 | 1,157488574 | 0,005199678 | 0 | 2,23068774 |
| mmu-miR-29b-1-5p | 4,070090149 | 2,909439386 | 1,160650764 | 0,008707755 | 0 | 2,235582464 |
| mmu-miR-29c-3p | 8,959877759 | 7,76193692 | 1,197940839 | 0,005199678 | 0 | 2,294119971 |
| mmu-miR-100-5p | 8,400795119 | 7,192062289 | 1,20873283 | 0,01642131 | 0,047619048 | 2,31134534 |
| mmu-miR-10a-5p | 4,503445929 | 3,219076868 | 1,284369061 | 0,012164432 | 0 | 2,435755057 |
| mmu-miR-23b-3p | 10,51192014 | 9,224143447 | 1,287776696 | 0,013263707 | 0 | 2,441515093 |
| mmu-miR-206-3p | 6,488324038 | 5,196554944 | 1,291769094 | 0,046068695 | 0,071428571 | 2,448280901 |
| mmu-miR-1839-5p | 5,49174691 | 4,195002287 | 1,296744623 | 0,008671936 | 0 | 2,456739045 |
| mmu-miR-183-5p | 7,590687287 | 6,185195031 | 1,405492256 | 0,019061027 | 0,047619048 | 2,649081549 |
| mmu-miR-34b-3p | 4,450178364 | 3,036687066 | 1,413491297 | 0,021790703 | 0,047619048 | 2,663810209 |
| mmu-miR-155-5p | 5,310874163 | 3,880687735 | 1,430186429 | 0,010251777 | 0 | 2,694815362 |
| mmu-miR-450a-5p | 6,02615757 | 4,563421177 | 1,462736393 | 0,031412382 | 0,130952381 | 2,756306631 |
| mmu-miR-152-3p | 8,959877759 | 7,496908011 | 1,462969747 | 0,01495242 | 0,023809524 | 2,756752497 |
| mmu-miR-10b-5p | 4,686689645 | 3,220750537 | 1,465939107 | 0,009625346 | 0 | 2,762432298 |
| mmu-miR-511-3p | 5,643626148 | 4,112654712 | 1,530971437 | 0,013263707 | 0 | 2,889803581 |
| mmu-miR-96-5p | 8,807562271 | 7,272356875 | 1,535205396 | 0,029288742 | 0,035714286 | 2,898296909 |
| mmu-miR-503-5p | 7,320550645 | 5,712330938 | 1,608219706 | 0,012955423 | 0 | 3,048753916 |
| mmu-miR-29a-3p | 12,03503326 | 10,40609123 | 1,628942035 | 0,005199678 | 0 | 3,092861081 |
| mmu-miR-148a-3p | 4,43165471 | 2,639059012 | 1,792595698 | 0,004329854 | 0 | 3,464376429 |
| mmu-miR-214-3p | 4,503445929 | 2,661605583 | 1,841840346 | 0,010251777 | 0 | 3,584670083 |
| mmu-miR-322-5p | 9,850337075 | 7,984796157 | 1,865540919 | 0,005199678 | 0,011904762 | 3,644045363 |
| mmu-miR-125b-5p | 11,38212187 | 9,513980339 | 1,86814153 | 0,003125173 | 0 | 3,650620065 |
| mmu-miR-29b-3p | 10,8597361 | 8,928592585 | 1,93114351 | 0,005199678 | 0,023809524 | 3,813573513 |
| mmu-miR-22-5p | 5,119835072 | 3,098862254 | 2,020972817 | 0,002791623 | 0 | 4,058573716 |
| mmu-miR-34c-3p | 5,177316658 | 3,142071906 | 2,035244753 | 0,008707755 | 0 | 4,098922614 |
| mmu-miR-199a-5p | 6,488324038 | 4,448632336 | 2,039691701 | 0,015015388 | 0 | 4,111576584 |
| mmu-miR-34c-5p | 8,370058024 | 6,303780829 | 2,066277195 | 0,008707755 | 0 | 4,188045729 |
| mmu-miR-150-5p | 4,273412736 | 2,043210774 | 2,230201962 | 0,003593691 | 0 | 4,69199658 |
| mmu-miR-142-3p | 8,279146324 | 6,041086462 | 2,238059862 | 0,008707755 | 0 | 4,717622114 |
| mmu-miR-199b-5p | 9,24573737 | 7,006918185 | 2,238819184 | 0,002791623 | 0 | 4,720105757 |
| mmu-miR-199a-3p | 9,327822452 | 7,076024884 | 2,251797568 | 0,005925948 | 0 | 4,762759065 |
| mmu-miR-34b-5p | 9,127390521 | 6,863955889 | 2,263434631 | 0,005925948 | 0,023809524 | 4,801331777 |
| mmu-miR-22-3p | 10,64411236 | 8,321986347 | 2,32212601 | 0,006152604 | 0,023809524 | 5,000685968 |
| mmu-miR-338-3p | 5,981980014 | 3,549928986 | 2,432051028 | 0,003125173 | 0 | 5,396601011 |
| mmu-miR-140-5p | 11,61358235 | 9,147820804 | 2,46576155 | 0,003593691 | 0 | 5,524184676 |
| mmu-miR-142-5p | 5,997924311 | 3,361698289 | 2,636226022 | 0,005199678 | 0 | 6,217032076 |
| mmu-miR-140-3p | 12,13282065 | 9,400171576 | 2,732649075 | 0,003243647 | 0 | 6,646749923 |
| mmu-miR-221-3p | 6,327451498 | 3,592373253 | 2,735078245 | 0,004329854 | 0,023809524 | 6,657950962 |
| mmu-miR-21-3p | 6,327451498 | 3,545685778 | 2,78176572 | 0,003125173 | 0 | 6,87693505 |
| mmu-miR-21-5p | 13,76230582 | 10,9247379 | 2,837567922 | 0,027666373 | 0,047619048 | 7,148140154 |
| mmu-miR-146b-5p | 6,658942451 | 3,77551449 | 2,88342796 | 0,005199678 | 0 | 7,379013526 |
| mmu-miR-205-3p | 2,561195207 | -0,630526514 | 3,191721721 | 0,009756727 | 0,023809524 | 9,137007386 |
| mmu-miR-222-3p | 5,968250638 | 2,736894296 | 3,231356342 | 0,003243647 | 0 | 9,39150482 |
| mmu-miR-205-5p | 10,30026232 | 3,218044797 | 7,082217519 | 0,025041295 | 0 | 135,5064317 |

This table lists all significantly differently regulated miRNAs of B78 melanoma samples by curcumin diet as analyzed with the Sure Print G3 miRNA V17.0 microarray chip (Agilent Technologies) based on miRBase 17.0. Displayed are the median of both groups for each miRNA, the difference between them (Δ log median), as well as adjusted *t*-test (*t*-test adjp.), the area under the curve (AUC) and the fold change median of the expression value.
